# Balloon kyphoplasty and additional anterior odontoid screw fixation for treatment of unstable osteolytic lesions of the vertebral body C2: a case series

**DOI:** 10.1186/s12891-018-2180-x

**Published:** 2018-07-27

**Authors:** Anna Voelker, Nicolas H. von der Hoeh, Christoph-Eckhard Heyde

**Affiliations:** University Hospital Leipzig, Department of Orthopedic, Trauma and Plastic Surgery, Liebigstrasse 20, 04103 Leipzig, Germany

**Keywords:** Osteolytic lesion C2, Metastasis dens, Anterior odontoid screw, Balloon kyphoplasty C2, Myeloma lesion dens axis, Cervical spine

## Abstract

**Background:**

Unstable osteolytic lesions of the occipitocervical junction are rare and may occur in hematological malignancy or vertebral hemangioma, among others. Different case reports have been published about vertebroplasty for treatment of spinal metastases of the upper cervical spine. Only few cases concern balloon kyphoplasty of C2. We present a consecutive case series including four patients with an osteolytic lesion of the dens axis and describe a technical note for balloon kyphoplasty of C2 and an additional anterior odontoid screw fixation.

**Methods:**

Four consecutive patients with an osteolytic lesion of the vertebral body of C2 were treated by anterior balloon kyphoplasty and additional anterior odontoid screw fixation of the dens axis. The radiological imaging showed a lytic process of the vertebral body C2 with no vertebral collapse but involvement of more than 50% of the vertebral body in all patients.

**Results:**

Two cases of potentially unstable osteolytic lesions of C2 by myeloma, one case with metastatic osteolytic lesion of C2 by adenocarcinoma of the colon and one patient with vertebral hemangioma located in C2 were presented to our clinic. In all cases, surgical treatment with an anterior balloon kyphoplasty of C2 and an additional anterior, bicortical odontoid screw placement was performed. Control x-rays showed sufficient osteosynthesis and cement placement in the vertebral body C2.

**Discussion:**

Anterior balloon kyphoplasty and anterior odontoid screw placement is a safe treatment option for large osteolytic lesions of C2. The additional odontoid screw placement has the advantage of providing more stabilization and may prevent late complications, like odontoid fractures. For patients with potentially unstable or large osteolytic lesions of the dens without spinal cord compression or neurological symptoms we recommend the placement of an anterior odontoid screw when performing a balloon kyphoplasty. Level of evidence: - IV: retrospective or historical series.

## Background

Metastatic lesions of the cervical spine are rare, especially at the occipitocervical junction (C0-C2). Metastatic spread occurs predominately at the thoracic and lumbal spine because of the better blood supply. Less than 1% of spinal metastasis occur at the craniocervical junction [1].

Nowadays, improved cancer treatment and longer life expectancy of tumor patients lead to an increasing number of this medical condition.

Most patients present with symptoms of heavy neck pain and a reduction of cervical motion. Neurological deficits are rare.

Myeloma is the most common underlying disease that leads to osteolytic lesions of the spine.

Bone destruction due to lytic lesions and generalized osteopenia may result in collapse of the vertebra [2]. Hence, pathological fracture of the vertebra body with neurological deficits, based on spinal cord compression, is the feared complication [2].

Literature describes different treatment options for treatment of C2 metastasis. Non- operative therapy includes immobilization, chemo- and external beam radiation therapy [3]. A variety of surgical procedures were published, including posterior instrumentation [4], open vertebroplasty by anterior approach [5–7], vertebroplasty by anterolateral approach either conventionally or percutaneously [8, 9], transoral vertebroplasty [10, 11], open dorsal vertebroplasty of the dens axis [12] and balloon kyphoplasty [13] .

One case report described balloon kyphoplasty and anterior screw fixation in a pathological fracture of the dens, induced by renal cell carcinoma [14]. Another case report described the treatment of a pathological fracture involving the odontoid process with radiofrequence thermoablation, cement augmentation and odontoid screw fixation [15]. Furthermore, Terraux et al. published a case report about balloon kyphoplasty and anterior screw fixation of odontoid fractures in two elderly patients [16]. Blondel et al. published a case series about anterolateral kyphoplasty in the management of cervical metastasis, including one case with a pathological fracture of C2 treated with kyphoplasty and an anterior screw [17]. In special cases unconventional procedures have been described. Bourgli used bone cement filling around a screw from C3 to odontoid in a case with anterior body collapse of C2 and a C1/C2 instability [18].

So far, no publication on balloon kyphoplasty and anterior odontoid screw fixation can be found for treatment of potentially unstable osteolytic lesions of C2.

We demonstrate a technical note for balloon kyphoplasty of C2 in combination with an anterior odontoid screw in two cases of osteolytic lesions in C2 by myeloma, in one case with metastatic osteolytic lesion by adenocarcinoma of the colon and in one case of large osteolytic process in C2 by hemangioma.

## Methods

Four Patients with unstable osteolytic lesion of the dens axis were treated with balloon kyphoplasty of C2 and an additional anterior, bicortical odontoid screw placement. All patients were presented to our clinic with neck pain. Imaging including x-ray, Computed tomography scan (CT-Scan) and Magnetic resonance imaging (MRI) of the cervical spine was performed. The definition of spinal instability was calculated by the Spine Instability Neoplastic Score (SINS). The radiological imaging showed a lytic process of the vertebral body C2 with no vertebral collapse but more than 50% involvement of the vertebral body in all patients. Surgical procedures were performed in the manner as described below.

### Technical note of balloon kyphoplasty of C2 and an additional anterior, bicortical odontoid screw placement

General anesthesia was performed with orotracheal intubation. The patient was placed on a radiolucent table in supine position with padding under the shoulders to induce reclination of the neck. Extension of the cervical spine was achieved by positioning the head in a horseshoe head rest. We used a gauze bandage in-between the jaw of the patient to obtain adequate AP view.

**Fig. 1:**
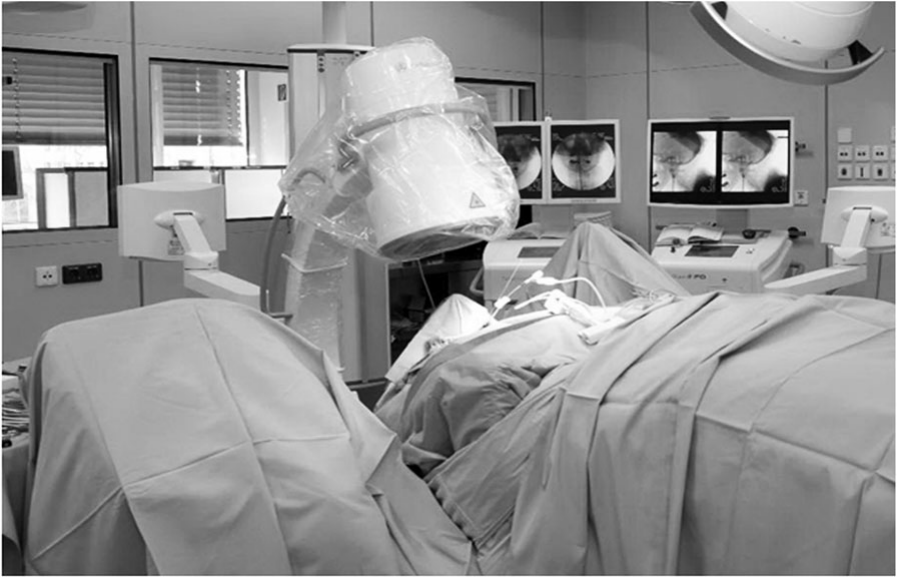
This picture shows the set-up in the OR with two c-arm X-ray units for anterior-posterior view, as well as for lateral view of the dens axis

Two c-arm X-ray units were used, one in anterior-posterior, and one in lateral view (Fig. 1).

After a free K-wire placement on the skin, to simulate the trajectory for the screw placement a unilateral transverse skin incision was made on the right side at the C5 level. The platysma was cut in longitudinal direction, as was the superficial cervical fascia. Atraumatic preparation was performed along the medial border of the sternocleidomastoid muscle to the middle cervical fascia. We approached the anterior surface of the spine after opening the middle cervical fascia and made a blunt dissection medial to the carotid artery, and lateral to the esophagus and trachea.

The inferior edge of the C2 vertebral body was palpated and a K-wire was drilled centrally into the odontoid process bicortically under biplanar fluoroscopy, using a cannulated drill bit system (2,7 mm) to prepare the screw hole bicortically.

**Fig. 2:**
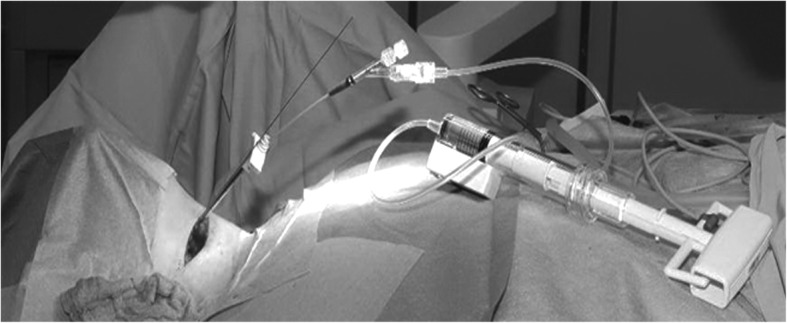
Shows the unilateral anterior transverse approach on the right side of the patient’s neck. K-wire is placed for anterior odontoid screw placement and the balloon for kyphoplasty into the dens axis

**Fig. 3:**
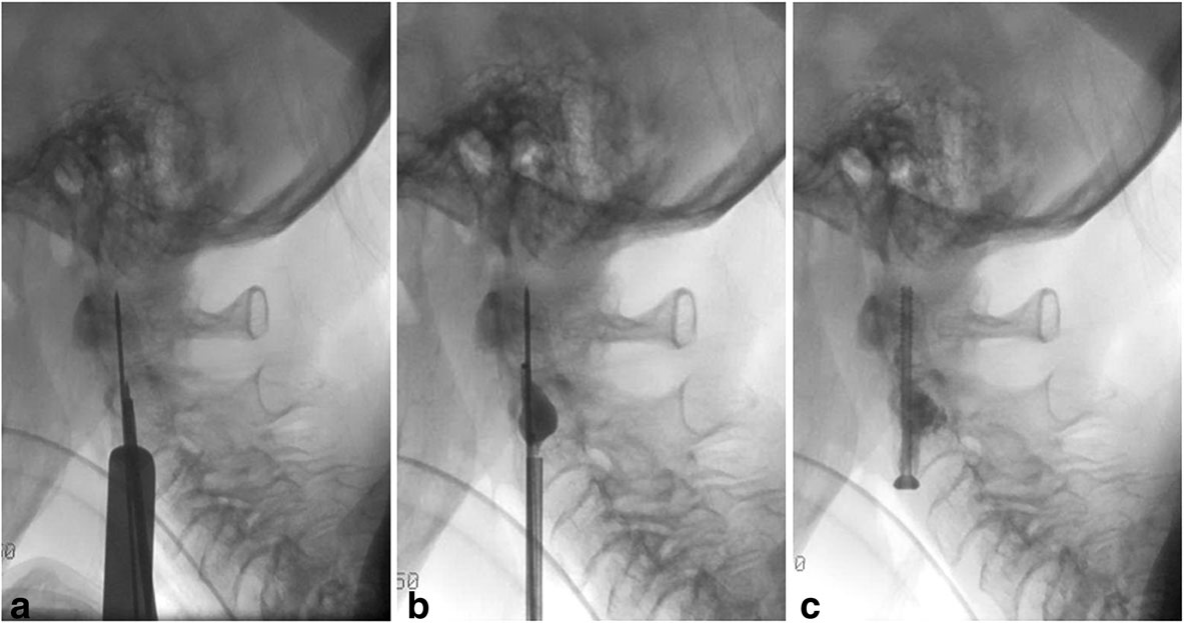
Shows step- by- step procedure under lateral fluoroscopic guidance. Picture A shows the placed K-wire in the odontoid process and the placed trocar for balloon kyphoplasty. Picture B shows the filled balloon within the osteolytic lesion of C2 and picture C shows the result after cement application in the osteolytic lesion, as well as the placed bicortical anterior odontoid screw

Following this, a second K-wire was placed on the anterior, inferior edge of C2, next to the screw hole in the area of the osteolytic lesion, to prepare the kyphoplasty working canal. Under fluoroscopy guidance, the K-wire was drilled in oblique and cranial direction into the osteolytic lesion, without penetrating the dorsal cortical bone of C2. Step by step, a cannulated drill bit system enlarged the entry point and the canal of the kyphoplasty trocar (2,7 mm and 3,5 mm drill bit). After removal of the second K-wire, the Jamshidi-Needle (*Kyphon*, Medtronic, USA) was placed into the osteolytic lesion under fluoroscopy guidance. A biopsy was performed with the hand drill for histopathologic investigation. Thereupon, the balloon was placed into the body of C2 through the working cannula and was expanded with contrast. (Fig. 2). Subsequently, the balloon was deflated, removed and the cavity was filled with cement, using the *Kyphon* cement fillers (Fig. 3). We used polymethylmethacrylate (PMMA) cement with high primary viscosity to prevent cement leakage. The used cement volume varied from case to case between 1,5 to 2,5 ml. Lastly, a cannulated 4.0 mm self-tapping screw was placed bicortically over the first K-wire. All single steps were performed with biplanar fluoroscopy guidance (Fig. 3). Example radiation exposure: area dose c-arm number one: 128,1 cGy*cm2; area dose c-arm number two: 95,7 cGy*cm2.

X-ray documentation showed satisfactory filling of the osteolytic lesion with cement and a good central screw placement. A soft collar was placed post operatively and the patient was monitored in the intensive care unit for 24 h after prompt extubation.

## Results

**Fig. 4:**
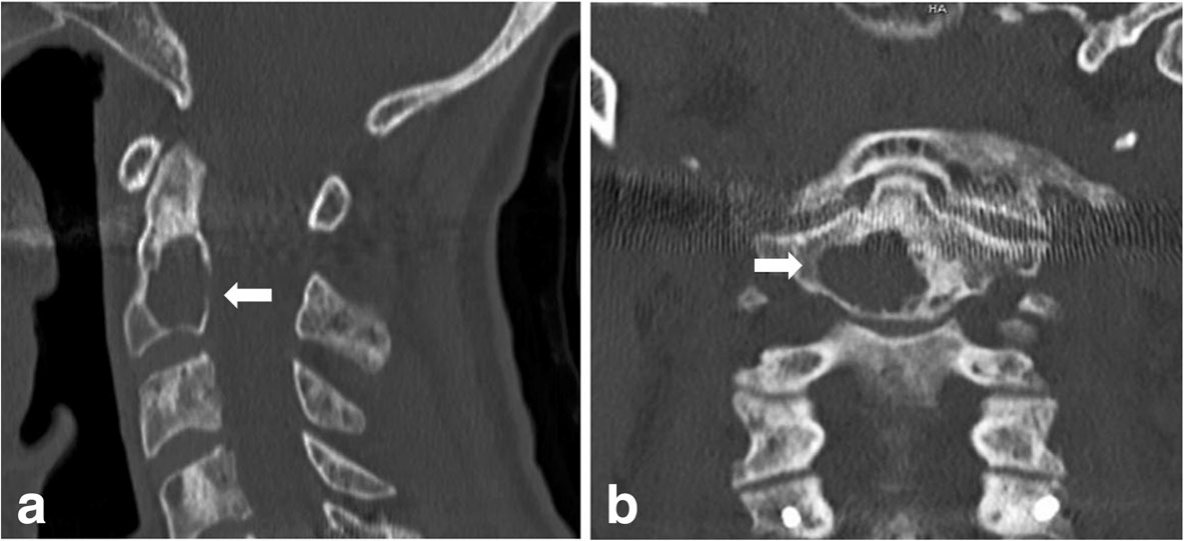
CT-scan in lateral (**a**) and coronal (**b**) view with instable osteolytic lesion (arrows) in the base of C2 (16 mm × 11 mm × 15 mm) (Case 1)

**Fig. 5:**
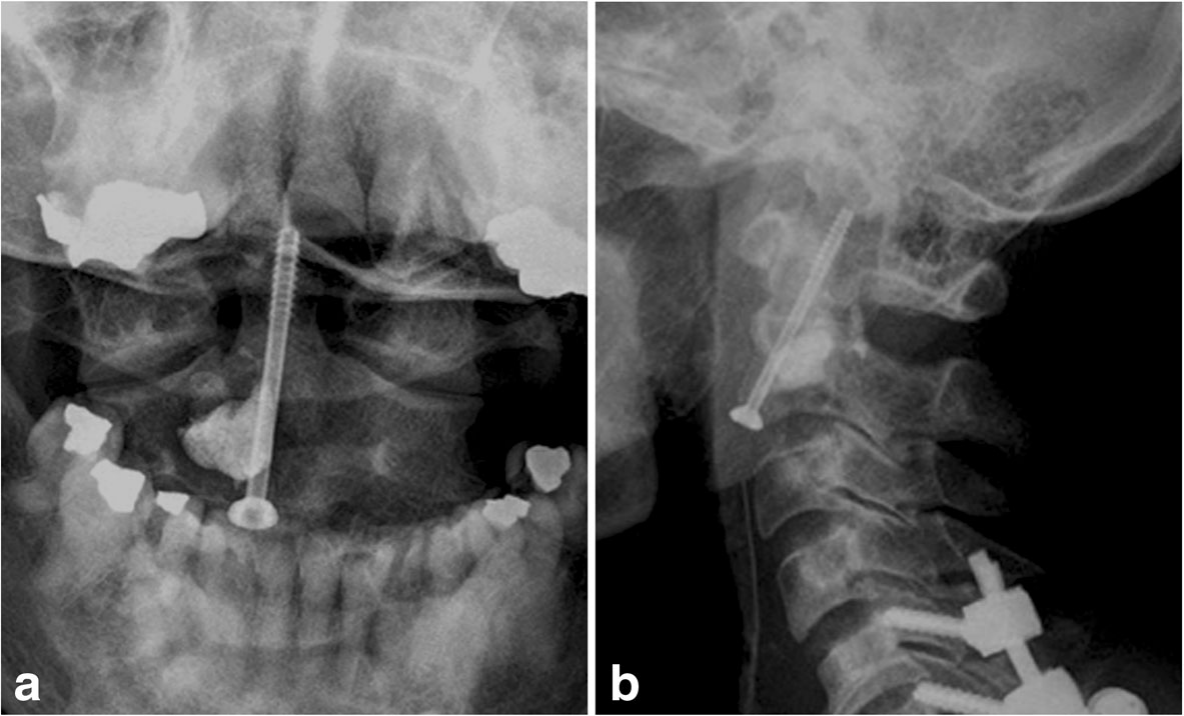
The postoperative x-ray shows cement in the base of C2 after balloon kyphoplasty and anterior odontoid screw (coronal view (**a**) and lateral view (**b**). Slight posterior cement leakage after kyphoplasty. Additional posterior instrumentation C5 to Th2 and laminectomy of C7 were performed for osteolytic lesion of C7 with compressed spinal cord (Case 1)

Case 1: A 76-year-old woman was presented to our clinic with severe neck pain and paresthesia in the left hand. Next to the multiple osteolytic lesions of the whole spine, CT-scan and MRI showed an unstable osteolytic lesion in C2 (16 mm × 11 mm × 15 mm) (Fig. 4) and an osteolytic lesion of C7 with destruction of the pedicle and with a spinal stenosis. First, we performed dorsal stabilization C5-Th2 with laminectomy of C7, based on mild neurological deficits in the left hand. The histopathological investigation confirmed suspected myeloma (Durie/Salmon Classification Stage III, Karnofsky Performance Score: 60%). Balloon kyphoplasty and anterior screw fixation of C2 was performed 2 weeks later. The patient recovered well. Neck pain diminished post operatively and x-rays showed good placement of the odontoid screw and cement in C2. Some cement leakage was detected posteriorly, however without neurological symptoms (Fig. 5). A soft collar was worn for 2 weeks postoperative. After wound healing, the patient was treated systemically by hematologist.

**Fig. 6:**
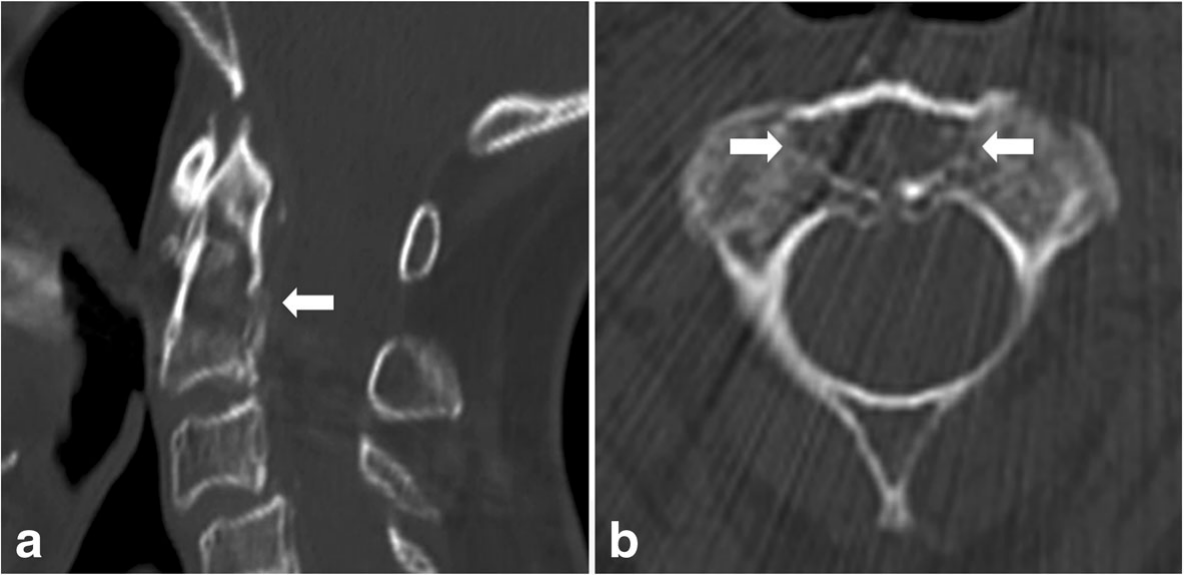
CT-scan in lateral (**a**) and axial view (**b**) of patient no. 2. A large osteolytic lesion in C2 (14 mm × 11 mm × 16 mm) is seen in the middle of the base and partially in the area of the odontoid process (arrows) (Case 2)

Case 2: An 82-year-old female patient with known myeloma (Durie/Salmon Classification Stage III, Karnofsky Performance Score: 70%) was presented in our outpatient clinic by her treating radiologist because of an osteolytic lesion in C2 (14 mm × 11 mm × 16 mm) (Fig. 6). The patient had a history of mild neck pain. The osteolytic lesion was detected during myeloma CT-scan staging. A MRI of the entire spine showed no further osteolytic lesions.

**Fig. 7:**
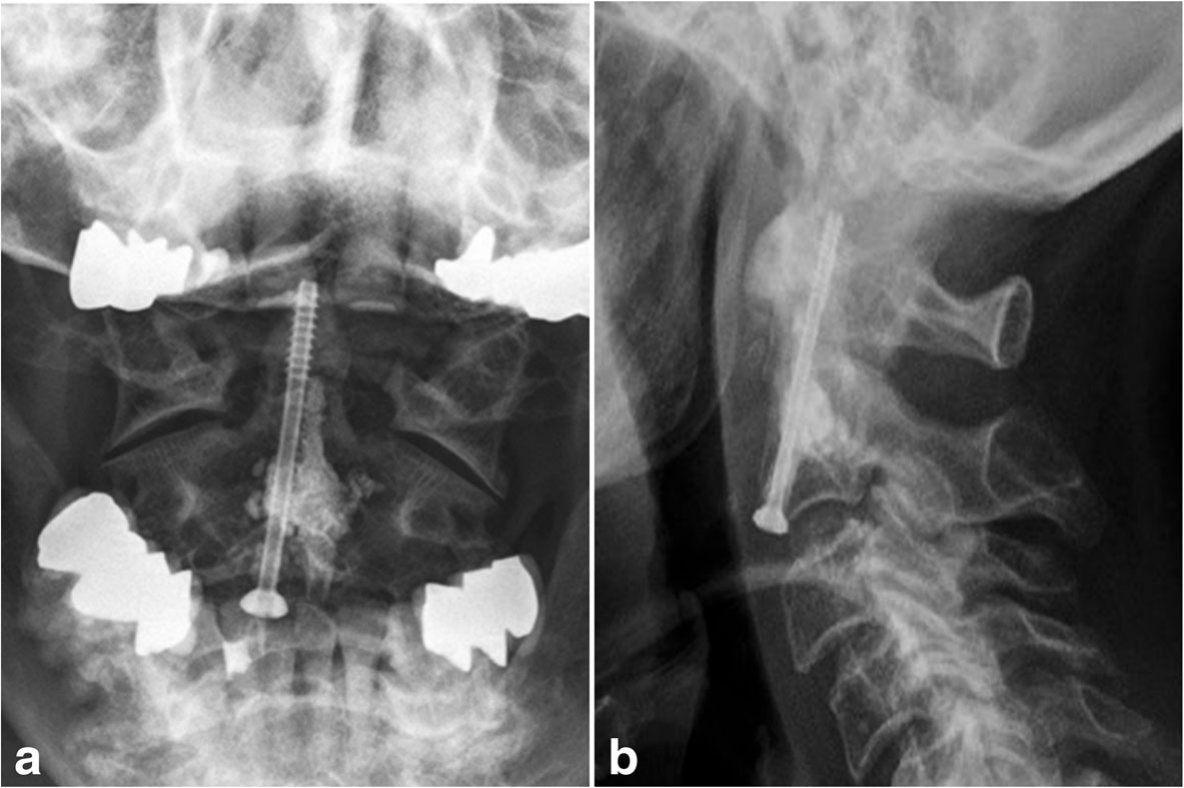
X-ray of the dens (**a**) and the lateral (**b**) cervical spine of the first patient shows favorable cement filling of the osteolytic lesion, in the base of the dens and the additional anterior odontoid screw (Case 2)

Surgery was performed in the same manner with balloon kyphoplasty of the osteolytic lesion of C2 and placement of an additional odontoid screw (Fig. 7). A soft collar was worn for 2 weeks after surgery. The patient recovered well. X-rays showed sufficient osteosynthesis and cement placement in C2. Further treatment of the myeloma was conducted by the hematologist.

**Fig. 8:**
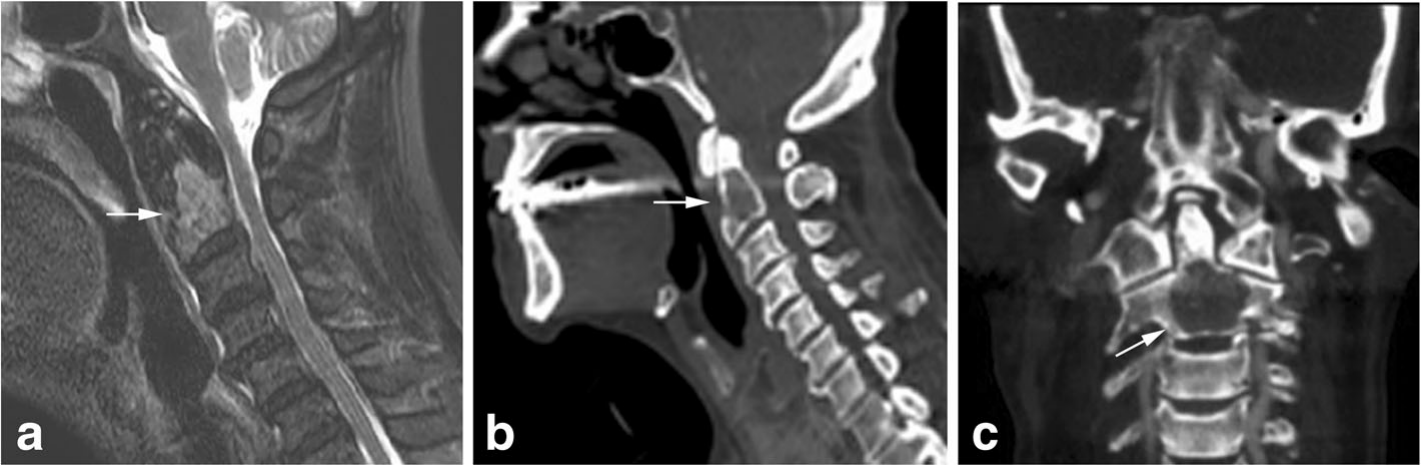
MRI of the cervical spine (**a**) shows a metastatic lesion in the vertebral body of C2 without spinal cord compression. The CT-scan in lateral and frontal view shows an osteolytic lesion (arrow) in the base of C2 (13,6 mm × 20 mm × 20,9 mm) in the same patient (**b** and **c**) (Case 3)

Case 3: A 78-year old man with a known history of adenocarcinoma of the colon (Karnofsky Performance Score: 60%) complained about a short history of severe neck pain. CT-scan of the cervical spine showed a large osteolytic process in the vertebra body of C2 (13,6 mm × 20 mm × 20,9 mm) (Fig. 8). MRI of the total spine ruled out further osteolytic lesions. Surgery with balloon kyphoplasty and additional odontoid screw was performed without complications. Following wound healing, radiotherapy of the dens axis was introduced (2,5 Gy five to six times per week, total dose 30 Gy).

**Fig. 9:**
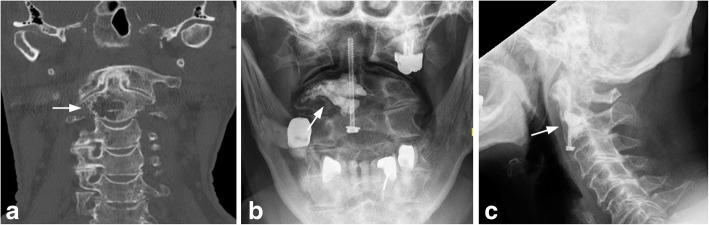
CT-scan of the upper cervical spine in lateral view with osteolytic lesion of C2 (arrow) of patient with vertebral hemangioma (**a**). Postoperative x-rays show a good filling of osteolytic lesion and the additional anterior odontoid screw (**b** and **c**) (Case 4)

Case 4: A 76-year old patient was presents to our clinic with a known osteolytic process inside the vertebra body of the dens axis (12,9 mm × 20,2 mm, 21 mm) (Fig. 9) by multiple hemangioma of the spine. In the patient’s history, an unstable osteolytic lesion of Th12 was stabilized with a screw-rod instrumentation. Radiological imaging with CT-scan and MRI of the cervical spine was done and surgery with balloon kyphoplasty and additional odontoid screw placement was performed. Control x-rays showed sufficient osteosynthesis and cement placement in the vertebral body C2 (Fig. 9). A soft collar was worn for 2 weeks after surgery.

## Discussion

Metastases, including osteolytic lesions of the craniocervical junction, are rare entities. Literature describes a variety of treatment procedures. The aim of treatment is to maintain stability for pain relief and to improve patients’ quality of life.

Chemotherapy and radiotherapy are useful options for conservative treatment.

Local radiotherapy of symptomatic bone lesions in myeloma patients reduce pain, yet it takes time until symptom relief sets in. Lee et al. reported the time span for symptom relief to be between 1 and 67 days (average time is 7 days) [19]. The study however does not describe improvement of bone stability through radiotherapy. Rief et al. showed that stability of bone occurred in one third of patients with osteolytic metastases of the thoracic and lumbar spine over a 6-month period after radiotherapy [20].

Rades et al. determined that multi-fraction radiotherapy shows a significantly better remineralization of the bone, than single fraction radiotherapy [21]. Remineralization begins 2 to 3 weeks after radiotherapy and the process continues to up to 2 months [22].

Treatment with radiotherapy is limited for patients with acute pain and in case of pathological fractures, because of the long duration of symptom relief and time needed for remineralization of the bone.

Different surgical therapies for cervical spinal metastases have been published. In general, patients with low risk myeloma have at least a 50% chance of surviving more than 10 years nowadays due to improved treatment [23]. One of our patients with myeloma was diagnosed initially on the basis of a biopsy taken from the tumor mass in C7 during the first surgery. The other patient had a known history of myeloma with a Durie/Salmon Classification Stage III and a Karnofsky Performance Score of 70%. The patient with known history of adenocarcinoma of the colon had a Karnofsky Performance Score of 60%. Goals of treatments of spinal metastasis are the pain reduction as well the upholding of the quality of life [24]. These were the reasons our decisions to operate was based on in all three patients with suspected tumor or known tumor disease.

For pathological fractures or large osteolytic lesions of the thoracic or lumbal spine vertebroplasty or balloon kyphoplasty are established procedures. Both techniques lead to adequate pain relief. In 1987 Galibert et al. published a case series of seven patients describing percutaneous acrylic vertebroplasty as a then new treatment in vertebral hemangioma instead of radiotherapy alone [25].

Khan et al. performed a pooled analysis of published case series of vertebral augmentation. Twenty-three studies, including balloon kyphoplasty and/ or vertebroplasty were evaluated. In all cases significant decrease in pain occurred shortly after the procedure. No difference in the extent of pain reduction between both procedures was found [26].

Only few studies about vertebroplasty and kyphoplasty in metastatic lesions of the dens axis were published.

Some case reports account for transoral approach for vertebroplasty of metastatic tumors in the upper cervical spine (C1 or C2), with or without combination of posterior stabilization. Patients treated transorally complained about pharyngeal discomfort and pain. Neck pain was reduced postoperatively [11, 27].

A CT guided percutaneous vertebroplasty technique of the upper cervical spine was described by Guo et al. [22]. They used a translateral approach between the carotid sheath and the vertebral artery for hemangiomas or metastatic lesions at C1-C3 in fifteen patients. Pain reduction could be achieved in all patients. Two patients had anterior cement leakage of the vertebra body. No vessel damage and no neurological symptoms occurred.

Kyphoplasty of C2 and C5 in a case of cervical metastases was published by Druschel et al. For this procedure, the typical anterior approach to the ventral cervical spine was used, like in the osteosynthesis of odontoid fractures [13]. Pain reduction after the procedure was good.

A meta-analysis comparing percutaneous vertebroplasty and balloon kyphoplasty for the treatment of single level vertebral compression fractures, carried out by Wang et al., showed balloon kyphoplasty to be superior. Balloon kyphoplasty has a higher volume of injected cement, better short- term pain relief, as well as a lower cement leakage rate than vertebroplasty. Longer operation time and higher material costs notwithstanding [28].

In our opinion the balloon kyphoplasty forms a more regular cavity and it is possible that some bone trabeculae are pressed against the wall through which it might be reinforced. On the other hand, ballooning should be performed carefully to ensure that the wall is not fractured. Additionally, the volume of the cement can be estimated due to the used volume for the balloon. The known lower cement leakage rate in balloon kyphoplasty is the main reason why we use this technique. Furthermore, the cavity created by the balloon leads to more application of cement and therefore to more stability of the vertebra body. In all four cases the main osteolysis was in the body of C2. Therefore, all screws had a good grip in the odontoid.

The complication rate after vertebroplasty and kyphoplasty in metastatic spinal diseases, as specified in literature, is about 4%. A systematic review about vertebroplasty and kyphoplasty for spinal metastases of C1 – C7 showed an asymptomatic cement leakage in 16% of the treated patients. About 4% of the patients had symptomatic complications, mainly mild odynophagia, occipital neuralgia, and one case with acute cerebellar and occipital infarction [29].

Another established method is combining a vertebroplasty of spinal osteolytic lesions associated with hematologic malignancies with radiation therapy to synergize the effects of both procedures on pain reduction and to minimize recurrence of local pain [30].

Occipitocervical posterior stabilization and, if necessary, decompression, is the treatment of choice in cases of kyphosis, instability and/ or collapse of the vertebra, as well as narrowing of neurological structures and neurological deficits in metastatic diseases of the upper cervical spine [4].

Anterior screw fixation is one of the common procedures in the operative management of type II odontoid fractures next to posterior C1/C2 stabilization and fusion. It provides stability, good fusion rates and preserves C1/C2 motion [31].

In our opinion, an additional anterior screw placement in the odontoid process provides more augmentation of the C2 vertebra.

Gumpert et al. demonstrated a demineralization at the cement surface after kyphoplasty for calcium phosphate cement, as a sign of remodeling of the cancellous bone [32]. This may pose a risk for subsequent pathological fracture after kyphoplasty. Vertebral fractures were also noticed after kyphoplasty in osteoporotic fractures of the lumbal spine [33]. We used PMMA cement for the kyphoplasties in our cases.

Odontoid fractures are the most common spine fractures in patients older than 80 years. Considering myeloma incidence increasing with age [34], most of these patients present a higher risk of traumatic dens fracture. A further aspect is the known osteoporosis in older women. Two of our patients were females over 70 years of age at the time of treatment. It is to be assumed, that both patients also suffer from osteoporosis.

As a result, we recommend an additional stabilization with an anterior odontoid screw, in case of kyphoplasty of metastatic lesions of the dens axis.

## Conclusion

Although the treatment of metastatic lesions of the dens is palliative, the life span of these patients increases due to better systemic therapy. Preserving functionality and reducing pain should be the ambition of treatment. Next to conservative treatment, surgical therapy may be necessary in cases of lytic lesions of the dens.

Our experience showed that balloon kyphoplasty and additional anterior odontoid screw fixation can be a good choice for treating patients with potentially unstable or large osteolytic lesions of the dens without spinal cord compression or neurological symptoms.

## Abbreviations

CT-Scan: Computed tomography scan
Gy: Gray (unit)
MRI: Magnetic resonance imaging
PMMA cement: Polymethylmethacrylate cement
SINS: Spine instability neoplastic score
